# Comparison of Vibrational Spectroscopic Techniques for Quantification of Water in Natural Deep Eutectic Solvents

**DOI:** 10.3390/molecules27154819

**Published:** 2022-07-27

**Authors:** Suha Elderderi, Pierre-Yves Sacré, Laura Wils, Igor Chourpa, Abdalla A. Elbashir, Philippe Hubert, Hugh J. Byrne, Leslie Boudesocque-Delaye, Eric Ziemons, Franck Bonnier

**Affiliations:** 1 EA 6295 Nanomédicaments et Nanosondes, Faculté de Pharmacie, Université de Tours, 31 Avenue Monge, 37200 Tours, France; suha.elderderimohmedabdelrhman@etu.univ-tours.fr (S.E.); igor.chourpa@univ-tours.fr (I.C.); 2 Department of Pharmaceutical Chemistry, Faculty of Pharmacy, University of Gezira, P.O. Box 20, Wad Madani 21111, Sudan; 3 Laboratory of Pharmaceutical Analytical Chemistry, CIRM, Vibra-Santé HUB, University of Liège (ULiege), Avenue Hippocrate 15, 4000 Liège, Belgium; pysacre@uliege.be (P.-Y.S.); ph.hubert@uliege.be (P.H.); eziemons@uliege.be (E.Z.); 4 EA 7502 Synthèse et Isolement de Molécules BioActives (SIMBA), Université de Tours, 31 Avenue Monge, 37200 Tours, France; laura.wils@etu.univ-tours.fr (L.W.); leslie.boudesocque@univ-tours.fr (L.B.-D.); 5 Department of Chemistry, College of Science, King Faisal University, P.O. Box 400, Al-Ahsa 31982, Saudi Arabia; bashir_gezira@yahoo.com; 6 Department of Chemistry, Faculty of Science, University of Khartoum, P.O. Box 321, Khartoum 11115, Sudan; 7 FOCAS Research Institute, Technological University Dublin, City Campus, Camden Row, Dublin 8, D08 CKP1 Dublin, Ireland; hugh.byrne@tudublin.ie

**Keywords:** label-free water quantification, natural deep eutectic solvent, partial least squares regression, attenuated total reflection infrared, Raman spectroscopy, near infrared spectroscopy

## Abstract

Vibrational spectroscopic techniques, i.e., attenuated total reflectance infrared (ATR-IR), near infrared spectroscopy (NIRS) and Raman spectroscopy (RS), coupled with Partial Least Squares Regression (PLSR), were evaluated as cost-effective label-free and reagent-free tools to monitor water content in Levulinic Acid/L-Proline (LALP) (2:1, mol/mol) Natural Deep Eutectic Solvent (NADES). ATR-IR delivered the best outcome of Root Mean Squared Error (RMSE) of Cross-Validation (CV) = 0.27% added water concentration, RMSE of Prediction (P) = 0.27% added water concentration and mean % relative error = 2.59%. Two NIRS instruments (benchtop and handheld) were also compared during the study, respectively yielding RMSECV = 0.35% added water concentration, RMSEP = 0.56% added water concentration and mean % relative error = 5.13% added water concentration, and RMECV = 0.36% added water concentration, RMSEP = 0.68% added water concentration and mean % relative error = 6.23%. RS analysis performed in quartz cuvettes enabled accurate water quantification with RMECV = 0.43% added water concentration, RMSEP = 0.67% added water concentration and mean % relative error = 6.75%. While the vibrational spectroscopic techniques studied have shown high performance in relation to reliable determination of water concentration, their accuracy is most likely related to their sensitivity to detect the LALP compounds in the NADES. For instance, whereas ATR-IR spectra display strong features from water, Levulinic Acid and L-Proline that contribute to the PLSR predictive models constructed, NIRS and RS spectra are respectively dominated by either water or LALP compounds, representing partial molecular information and moderate accuracy compared to ATR-IR. However, while ATR-IR instruments are common in chemistry and physics laboratories, making the technique readily transferable to water quantification in NADES, Raman spectroscopy offers promising potential for future development for in situ, sample withdrawal-free analysis for high throughput and online monitoring.

## 1. Introduction

In recent years, industry and research facilities have striven to accelerate the transition towards green processes and technologies. The field of industrial chemistry aims to reduce the negative environmental impact of chemical residues by utilising bio-renewable and biodegradable resources in a sustainable manner. In this context, Natural Deep Eutectic Solvents (NADES) hold great promise as a replacement for toxic organic solvents [1,2,3]. NADES have unique solvent properties, such as high extraction ability and high solubilisation strength for a wide range of organic and inorganic compounds [2,4,5,6]. In addition to being easily prepared, cost effective and easily tuneable for specific applications, they can outcompete other solvents in terms of extraction rates and efficacy [6,7,8]. NADES are increasingly studied for numerous reported applications including analytical chemistry [4], organic synthesis [9], biotechnology [10,11], electrochemistry [12], nanotechnology [13,14,15], energy [16], water remediation [17], cosmetics and pharmaceutics [18,19] and food industries [20,21,22].

NADES are viscous solvents [7], which is considered to be a major barrier in analytical chemistry applications [1,7,23,24]. However, the constituent compounds of NADES are often hygroscopic by nature and although, upon preparation, samples contain an initial water content <1% *w*/*w*, the controlled addition of water can be employed to systematically decrease the viscosity and improve solvation and mass transfer operations, therefore ensuring maximum efficiency during extraction [23,24,25]. According to the type of NADES, water molar concentration ratio is critical for certain applications, such as enzyme reactions and dissolution of compounds in cosmetics and pharmaceutical areas [1,7]. Moreover, the polarity of the NADES increases with water content, strongly affecting their solubilisation capacity depending on the nature of solutes [7]. However, increased water content can weaken the interactions between the NADES and the target compounds, as well as the interactions between the components of the NADES themselves, until complete disruption occurs [24]. Therefore, the control of water content and the stability of NADES-formulated products remains one major bottleneck to more extensive industrial use [1,6,7]. Quantification of water content is therefore essential to ensure the reproducibility of experiments, especially after a storage period, because NADES tend to accumulate water from the ambient air, after water was purposely added for a specific application for optimal use, or water can also derive from a biomass (plants, algae) during extraction [1,6,7].

The techniques commonly used for water quantification are Karl Fisher (KF) titration and the gravimetric method. KF titration is the gold standard method for quantification of residual water with numerous examples in organic solvents [26], plant extracts [27] or in food [28,29]. Despite the sensitivity of the method, the large volumes of reagent and solvent consumed for titration (especially for high water content) and the time requirements for analysis of large sample cohorts [30] motivate the development of alternatives. KF titration is known to determine water selectively by a chemical reaction [31]. Although it is considered the most accurate for determining water content, is to be noted that values obtained may depend somewhat on experimental conditions of titration (solvent utilised, temperature) [29]. The gravimetric method is the simplest, solvent-free, cost-effective technique, which measures the weight loss of a sample due to water evaporation while drying under heating. However, a lack of repeatability in results is observed due to thermal decomposition of the sample or for volatile samples [30]. It has also been demonstrated that, in materials with high viscosity, the formation of a rubbery matrix during drying makes water diffusion and evaporation difficult, leading to underestimation of moisture levels [29,32].

In the context of the principles of Green Analytical Chemistry (GAC) [33], environmentally-friendly techniques that provide rapid and accurate water quantification in NADES are needed. Vibrational spectroscopy, i.e., near-infrared spectroscopy (NIRS), mid-infrared spectroscopy (MIRS) or Raman spectroscopy (RS), includes non-destructive, non-invasive, label-free, reagent-free and cost-effective spectroscopic techniques that can be applied to rapidly probe the chemical composition of samples with minimum or no sample preparation. Vibrational spectroscopy has been widely used for chemical characterisation and molecular imaging of pharmaceuticals [34,35], biological tissues [36,37] or even subcellular analysis [38,39,40]. Moreover, it is used as a powerful quantitative analysis tool for complex solutions such as human serum [41], chemotherapeutic solutions [42,43], pharmaceuticals solid dosage forms [44,45,46] or cosmetic products [47].

Water quantification, or moisture analysis, using vibrational spectroscopy has been reported in the literature for MIRS in lubricants [48,49] or milk [50]; for RS in trace water quantification in glass [51,52] and recently for moisture content determination in flour or pasta samples [53,54]; and for NIRS in food [55] as a PAT (process analytical technology) tool for moisture content determination during freeze-drying processes [56,57], in lubricants [49], organic solvents [58] or absolute water content of minerals [59], water content in skin [60,61,62] and pharmaceutical pellets [63]. Recently, Elderderi et al. reported the first demonstrations of attenuated total reflectance MIRS (ATR-IR) [64] and RS [65,66] for water quantification using glycerol-based NADES as models, i.e., Betaine:Glycerol (1:8, mol/mol), Choline Chloride:Glycerol (1:2, mol/mol) and Glucose:Glycerol (1:3, mol/mol), each with systematically varying added water concentrations in the range of 0% *w*/*w* to 40% *w*/*w*.

The intrinsic water content of NADES is typically >1% *w*/*w*, and therefore may not require the high sensitivity of the KF titration technique. Spectroscopic analyses offer reagent-free, and therefore greener, techniques which are potentially field deployable in the industrial environment. The present study aims to undertake a direct comparison of the potential of ATR-IR spectroscopy, RS and NIRS for monitoring water content in Levulinic Acid/L-Proline (LALP) (2:1, mol/mol) NADES as a model case study. While benchtop systems have been used for the three techniques, a handheld device for NIRS has also been included to determine quantitative performance for the commercialised compact instruments, which may be more easily field deployable. The performance of each instrument coupled to Partial Least Squares Regression (PLSR) is quantified in terms of linearity of the regression between the measured and predicted concentrations (R^2^), Root Mean Square Error of Cross-Validation (RMSECV), Root Mean Square Error of Prediction (RMSEP) and accuracy of the predicted concentration expressed by the % relative error compared to the target (true) concentration.

## 2. Materials and Methods

### 2.1. Reagents

L-Proline (Acros Organics™, 99%, Geel, Belgium) and Levulinic Acid (Acros Organics™, 98%, Geel, Belgium) were purchased from Fisher scientific SAS (Illkirch, France). Water was purified using a Milli-Q system (Millipore Corporation, Bedford, MA, USA).

### 2.2. Preparation of Levulinic Acid/L-Proline (LALP) NADES Samples

**Figure 1:**
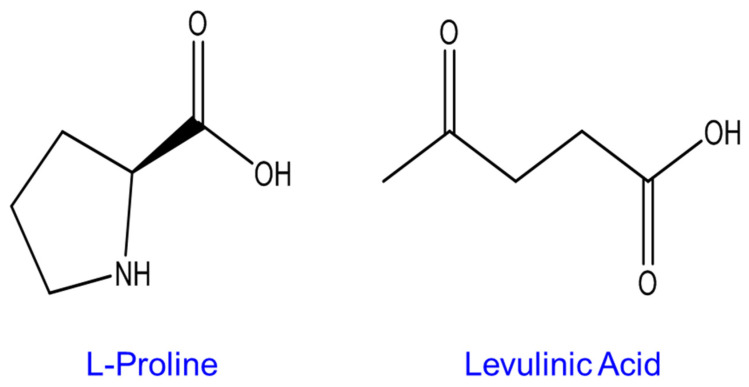
Chemical structures of L-Proline and Levulinic Acid.

The NADES model selected for this study has been prepared from hydrogen bond donor (HBD) Levulinic Acid (LA) and Hydrogen bond acceptor (HBA) L-Proline (LP) (Figure 1). The resulting NADES Levulinic Acid/L-Proline (LALP) was prepared according to the heating and stirring protocol described by Dia et al. [1]. Briefly, LA and LP were mixed in a 2:1 molar ratio, and then heated at 70 °C under magnetic stirring for 1.5 h until a homogenous colourless phase was formed. NADES intrinsically contain initial water content which, in this case, has been determined to be 1.07+/−0.08% *w*/*w* from 3 Karl Fisher titrations performed on 3 different days. Therefore, the present study is designed based on the standard addition protocol [66,67]. Therefore, results throughout the manuscript are presented as % *w*/*w* added concentration of water in NADES samples. For the purpose of the study, 9 samples with % *w*/*w* added water concentrations—C1 (0% *w*/*w*), C2 (≈0.99% *w*/*w*), C3 (≈2.4% *w*/*w*), C4 (≈4.76% *w*/*w*), C5 (≈6.98% *w*/*w*), C6 (≈9.09% *w*/*w*), C7 (≈16.67% *w*/*w*), C8 (≈23.07% *w*/*w*) and C9 (≈28.57% *w*/*w*)—were prepared by weighing. The exact mass of added water weighed for each sample has been used for the calculations and statistical analysis. For the purpose of the study, 10 g of NADES was prepared for each water concentration with an analytical balance with 0.1 mg precision, resulting in errors in reference concentration as low as 0.002%. Considering the range of added water concentration, from 0% *w*/*w* to 28% *w*/*w*, it is assumed these errors have no effect on the PLSR models (see Section 2.3). Five replicate sets of NADES (SET_01, SET_02, SET_03, SET_04 and SET_05) each consisting of nine concentrations (C1–C9), a total of 45 samples, were prepared independently and analysed for statistical purposes.

### 2.3. Data Collection

#### 2.3.1. Attenuated Total Reflectance (ATR-IR) Spectroscopy

ATR-IR spectra were acquired using a Frontier Fourier transform infrared (FTIR) spectrometer (Perkin Elmer, Villebon-sur-Yvette, France) equipped with a Quest single reflection diamond attenuated total reflectance (ATR) accessory (Specac, Orpington, UK). The spectral range was set between 4000 and 400 cm^−1^ with spectral resolution of 4 cm^−1^. A drop ≈100 µL was deposited directly onto the diamond surface and spectroscopic measurements were performed immediately. Prior to sample measurement, a background spectrum was recorded in the air (4 averaged scans) and the sample spectrum (4 averaged scans) was automatically ratioed with it via software (Spectrum, Perkin Elmer), effectively normalising it to a maximum reflectance of 1 (100%). The data were further processed by software to express the spectrum in terms of sample absorbance. For each sample, 3 deposits have been measured and 3 spectra per drop have been collected. Ultimately, 9 spectra were recorded from each sample, capturing inter- and intra-variability during measurements. Spectra from pure compounds have also been collected using similar parameters. The entire operation to analyse 1 drop, including cleaning the ATR crystal, collection of the background, and collecting the IR spectrum from the sample, takes less than 30 s.

#### 2.3.2. Benchtop Near Infrared Spectroscopy (NIR-B)

NIR-B spectra were acquired using a Multipurpose Fourier transform near infrared spectrophotometer (MPA, Bruker Optics, Ettlingen, Germany) equipped with a TE-InGaAs detector. Spectra were collected with the internal transmission module. The spectral range was 12,800–4000 cm^−1^ (781–2500 nm) and the spectral resolution 8 cm^−1^. Prior to sample measurement, a background spectrum was recorded in the air (32 scans) and the sample spectrum (32 averaged scans) was automatically ratioed with it via software (Opus 6.5, Optics Bruker), normalising the spectrum to a maximum of 1. The data were further processed to express the spectrum in terms of absorbance. Samples were directly scanned in 1 mL shell type glass vials. For each sample, the vial was placed in the instrument and 3 spectra collected, the vial was then removed and the next concentration was analysed. Once the 9 samples were analysed, the entire operation was repeated twice to capture inter- and intra-variability during measurements, notably due to repositioning of the vial. Spectra from pure compounds were also collected using similar parameters.

#### 2.3.3. Handheld Near Infrared Spectroscopy (NIR-H)

NIR-H spectra were acquired using a handheld dispersive NIR transmission spectrometer (NIR-M-T1, Innospectra Corp., Hsinchu, Taiwan). The spectral range was 11,111 to 5882 cm^−1^ (900–1700 nm). The lamp was turned on for 1 h before starting the analysis to reach a stable detector temperature, from whence the lamp remained lit up during the analysis of the whole series. However, the device was shut down and cooled to room temperature between each validation series. Prior to sample measurement, a background spectrum was recorded in the air (32 scans) and the sample spectrum (32 averaged scans) was automatically ratioed with it via software (ISC SDK GUI v3.7.2, Innospectra). The data were further processed to express the spectrum in terms of absorbance. Samples were directly scanned in 1 mL shell type glass vials. For each sample, the vial was placed in the instrument and 3 spectra were collected, the vial was then removed and the next concentration was analysed. Once the 9 samples were analysed, the entire operation was repeated twice to capture inter- and intra-variability during measurements, notably due to repositioning of the vial. Spectra from pure compounds were also collected using similar parameters.

#### 2.3.4. Benchtop Raman Microscope (Raman-B)

Raman-B spectra were collected using a Labram spectrometer (Horiba Jobin-Yvon, Palaiseau, France) equipped with a 691 nm laser source delivering ~10 mW at the sample. A macro-sampling holder, consisting of a cuvette holder attached to the turret of the microscope (Horiba Jobin-Yvon, Palaiseau, France), was employed. The laser coming out of the turret was reflected by a 45° mirror and directed through the quartz cuvette containing 500 µL of the solution. This set-up provides maximum reproducibility between measurements and the risk of any photothermic damage is minimised because the laser is not tightly focused, although it requires larger volumes to perform the analysis. The spectral range was set between 150 and 4000 cm^−1^, resulting in a spectral resolution of ~2.5 cm^−1^, achieved using 300 lines/mm grating. Five accumulations of 1 s were taken for each spectrum. For each sample, the quartz cuvette was placed in the sample holder and 3 spectra were collected, the cuvette was then removed, the operation repeated twice and the next concentration analysed. Ultimately, 9 spectra were recorded from each sample, capturing inter- and intra-variability during measurements, notably due to repositioning of the quartz cuvette. Spectra from pure compounds were also collected using similar parameters. The entire operation to analyse 1 cuvette takes less than 20 s.

### 2.4. Data Analysis

For consistency, the size of the data sets has been kept identical for all spectroscopic techniques, i.e., 5 sets of NADES × 9 concentrations × 9 spectra recorded for each concentration = 405 spectra.

Data pre-processing and analysis were performed using MATLAB^®^ (The Mathworks, Natick, MA, USA). *Pre-processing:* In analytical applications of vibrational spectroscopy, it is quite common to apply pre-processing techniques such as background/baseline correction, normalisation and/or derivatisation [68]. For the data collected using ATR-IR, NIR-B and NIR-H, it has been observed that the best outcome was achieved using raw spectral data, which have been normalised to the blank reference and converted to absorbance. This is consistent with a previous study by Elderderi et al. [64] for water quantification in 3 glycerol-based NADES using ATR-IR. Therefore, in the present study, analysis of data from these techniques was performed without any pre-processing.

Consistent with previous studies by Elderderi et al. [65,66], raw Raman spectra did not deliver the optimal outcome for Raman-B; therefore, for the purpose of this study aiming at illustrating the best achievable quantitative performance, a pre-processing protocol has been applied.

To minimise variability due to any baseline of the spectra, a Rubberband (RB) correction has been applied. RB is an algorithm which estimates a piecewise polynomial baseline. Firstly, a set of support points is determined such that the region below these points form a convex hull and, secondly, polynomial curves are estimated between each support point, which is then subtracted from the spectra [69,70]. In the current study, the polynomial order was set to 1 to avoid overcorrection of spectra, considering the quality of recorded spectra [71]. To compensate for any disparity in intensity levels, a vector normalisation (VN), i.e., calculation of the ratio of spectra to their respective Euclidian norms, has been applied to rescale spectra [72].

*Partial Least Squares Regression (PLSR):* PLSR is a supervised multivariate method widely used to extract quantitative information from spectral data sets [73]. In this study, the technique is applied to the data to construct a linear model between variations in spectral features and the systematically varied water concentrations.

The statistical relevancy of the quantitative analysis performed was evaluated through cross-validation procedures with a three-way splitting of the data into calibration, validation and test sets [74]. For the purpose of the study, 5 sets of data were prepared and analysed (SET_01, SET_02, SET_03, SET_04 and SET_05). Spectral data collected from SET_02 and SET_04 (n = 18 samples) were used as training sets for the calibration and validation steps of the quantitative models. Subsequently, 2/3 of the samples (n = 12) of the training set were randomly selected as the calibration set, and the remaining 1/3 of samples (n=6) were used as validation set. A 100-fold iteration was implemented to evaluate the stability of the analysis with multiple random combinations of calibration/validation sets. Notably, the validation set is used to select the optimal number of latent variables (LVs). SET_01, SET_03 and SET_05 (n = 27 samples) were used as the independent test samples to be determined in the predictive model (i.e., unknown to PLSR model). Although the concentration in the test samples is known, these values are not used to construct the PLSR model, but only at a later stage to assess the performance of the model. The output of this model provides information to evaluate PLSR using the linearity of the regression between the measured and predicted concentrations (R^2^), Root Mean Square Error of Cross-Validation (RMSECV) calculated from the validation datasets (i.e., SET_02 and SET_04) and Root Mean Square Error of Prediction (RMSEP) calculated from the test set (i.e., SET_01, SET_03 and SET_05). The accuracy of the prediction concentration expressed by the % relative error compared to the target (true) concentration and regression vectors, which represents the variables (wavenumbers), was used to construct the predictive model. In this study, predicted concentrations, RMSECV and RMSEP are expressed as % *w*/*w* added water concentration.

## 3. Results and Discussions

### 3.1. Construction of Predictive Models and Spectral Characterisation

#### 3.1.1. ATR-IR Spectroscopy

**Figure 2:**
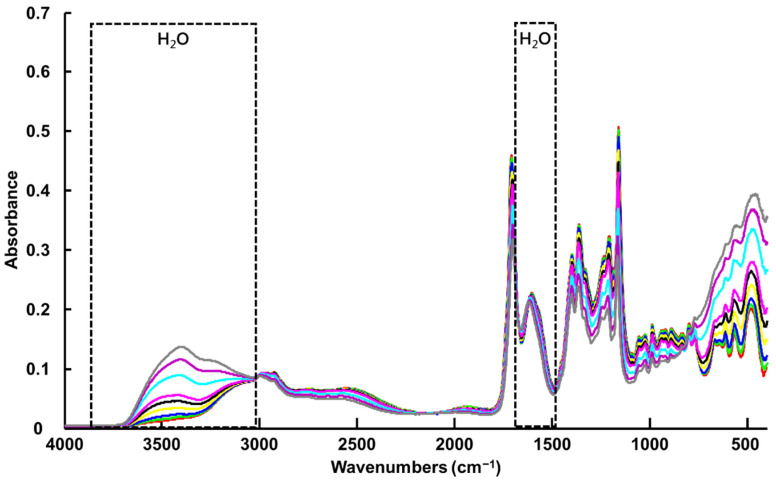
Mean raw ATR-IR spectra collected from LALP. Concentrations for added water (% *w*/*w*) are respectively ~ 0% (red), 0.99% (green), 2.4% (blue), 4.7% (yellow), 6.9% (black), 9.1% (magenta), 16.7% (cyan), 23% (purple) and 28% (grey).

**Figure 3:**
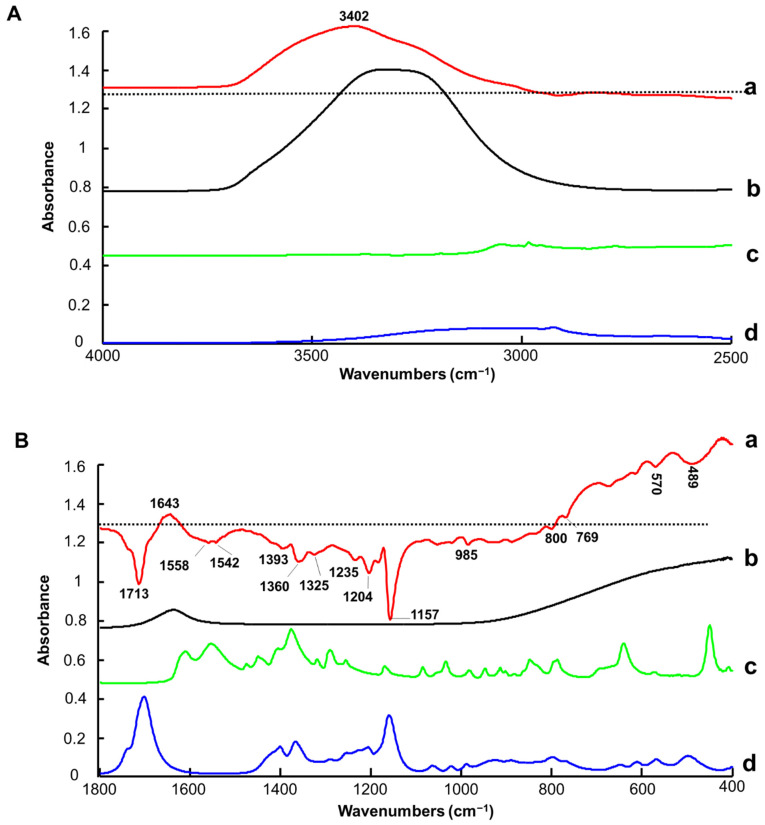
First regression coefficient from PLSR of ATR-IR (dotted line indicates the zero baseline) (a), water spectrum (b), L-Proline (c) and Levulinic Acid (d) in the spectral range 4000–2500 cm^−1^ (**A**) and the spectral range 1800–400 cm^−1^ (**B**). Spectra are offset for clarity.

The vibrational spectrum of water recorded using the FTIR spectrometer equipped with an ATR accessory exhibits two strong features, a broad band in the spectral range 3700 to 3000 cm^−1^ with a maximum value of ~3350 cm^−1^, assigned to symmetrical and asymmetrical stretching of the H_2_O molecules, and a sharp band with lower intensity at ~1640 cm^−1^, assigned to scissoring/bending [75,76] (Figure 2 and Figure 3A(b),B(b)). These observations are consistent with previously reported data by Elderderi et al. that were collected with the same system [64]. The contribution of water features can be clearly seen in the high wavenumber region of the ATR-IR spectra collected from LALP NADES samples with increasing added % *w*/*w* water concentration (Figure 2).

In the fingerprint region, L-Proline and Levulinic Acid display numerous sharp peaks overlapping with the contribution of water, such that the feature at ~1640 cm^−1^ is not discernible between the strong L-Proline and Levulinic Acid features at ~1600 and ~1750 cm^−1^, respectively (Figure 3B(c,d)). In the ATR mode, the evanescent wave of the IR source probes a fixed sample depth; therefore, the systematic variation of the water content of the NADES samples results in a systematic increase in the water contributions at ~3350 cm^−1^, and a concomitant, anticorrelated decrease in NADES features, most notably across the fingerprint region.

Figure S1A shows the PLSR plot obtained from the validation sets (SET_02 and SET_04) using four latent variables (LV), with the optimal number selected based on the lowest RMSECV obtained with the validation sets (Figure S1B). The RMSECV of 0.27 +/− 0.17% *w*/*w* added water concentration and the R^2^ value of 0.9990 +/− 0.0016 indicate good fitting of the data over the concentration range analysed (Figure S1A). This is confirmed visually by the tight distribution of predicted concentrations around the regression line, also suggesting high reproducibility of measurements performed and hence a low intra- and inter-set variability.

The first regression coefficient (Figure 3A(a),B(a)) highlights the positive contribution of water in the high wavenumber region, evidenced by the strong band with a maximum value of 3402 cm^−1^ (Figure 3A(a)) and in the fingerprint region by the band at 1643 cm^−1^ (Figure 3B(a)). The negative bands in the fingerprint region are assigned to the LALP compounds that have intensities which are anticorrelated with water content. The features at ~1713 (C-O stretching vibration), 1393 (H-C-H scissoring vibration), 1360 (C-H bending vibration), 1235 cm^−1^ (C-H twisting + OH twisting), 1204 (C-C-H bending vibration [77]), 1157 (C-O-H in plane bending), 985 (C-O-H bending vibration), 800 (whole molecule bend), 769 (C-H twisting), 614 (wagging O-H), 570 (Torsion (C-C-C-C) whole molecule) and 489 cm^−1^ (torsion in whole molecule) can be assigned to Levulinic Acid (Figure 3B(d)) [78]. The broad band with 2 maxima at 1558 and 1542 cm^−1^ (NH in plane bending) and the weak feature at 1325 cm^−1^ correspond to L-proline contributions (Figure 3B(c)). Other L-Proline features at 1609 (C=O stretching vibration), 1289 (CH in plane bending vibration) and 1375 cm^−1^ (OH in plane bending) do not clearly appear in the regression coefficient due to overlaps with strong Levulinic Acid features (Figure 3B(d)) [79].

Clearly, while the water bands play a key role in both the high wavenumber and fingerprint regions for the construction of the quantitative model, variation of features specific to NADES constituents (i.e., Levulinic Acid and L-Proline) also contributes significantly to the PLSR analysis, most prominently in the fingerprint region.

#### 3.1.2. Benchtop NIR Spectroscopy (NIR-B)

**Figure 4:**
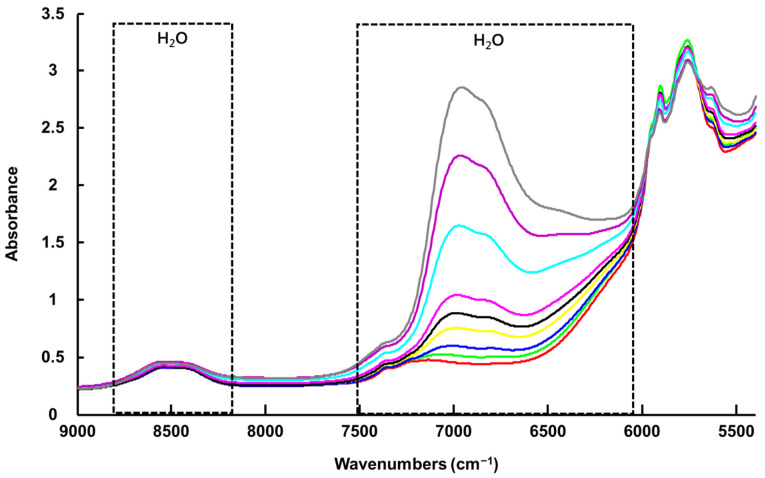
Mean raw NIR-B spectra collected from LALP. Concentrations for added water (% *w*/*w*) are respectively ~ 0% (red), 0.99% (green), 2.4% (blue), 4.7% (yellow), 6.9% (black), 9.1% (magenta), 16.7% (cyan), 23% (purple) and 28% (grey).

**Figure 5:**
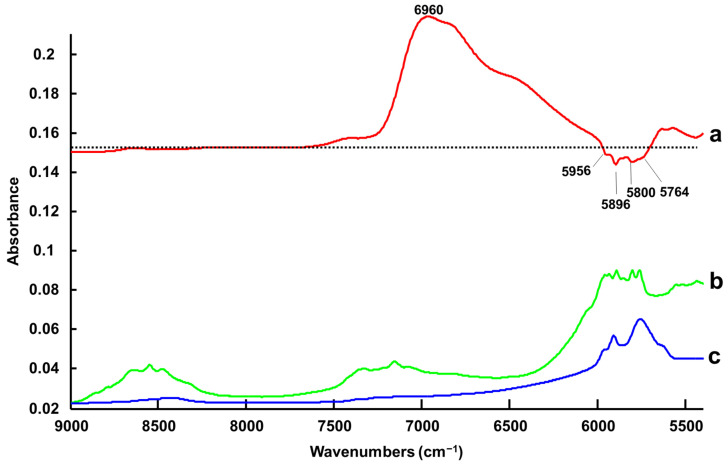
First regression coefficients from PLSR of NIR-B (dotted line indicates the zero baseline) (**a**) compared to the spectra of L-Proline (**b**) and Levulinic Acid (**c**). Spectra have been offset for clarity.

**Figure 7:**
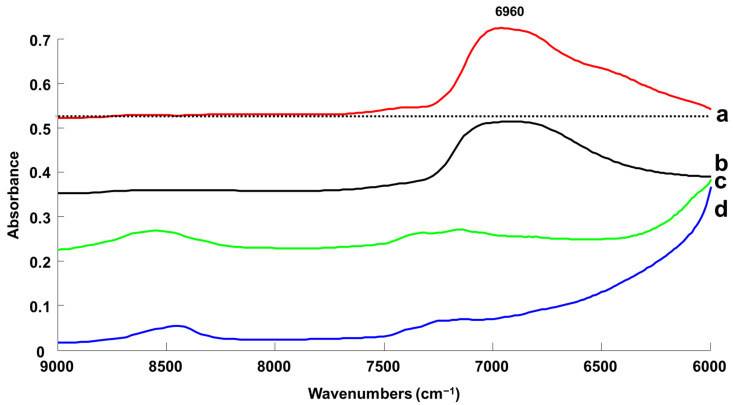
First regression coefficient of PLSR of NIR-H (dotted line indicates the zero baseline) (**a**), water spectrum (**b**), L-Proline (**c**) and Levulinic Acid (**d**). Spectra have been offset for clarity.

Due to saturation of the signal, no experimental spectrum from water could be recorded to compare with the regression coefficient from PLSR (Figure 5). Nevertheless, based on the literature, the water spectrum exhibits three main features in NIRS, a strong band around 5200 cm^−1^, assigned to a combination of OH stretching and bending overtones, another at 7000 cm^−1^, assigned to the first overtone of OH stretching, and another at 8500 cm^−1^, assigned to the combination of OH bending and first overtone OH stretching [55,76] (Figure 4 and Figure 7b). Although NIR spectra collected with a benchtop system (NIR-B) cover the spectral range 12,500–4000 cm^−1^, the first band at 5200 cm^−1^ could not be used, as the high-water concentrations in samples resulted in saturation of the signal. PLSR analysis was therefore only applied to the region 9000–5400 cm^−1^, due to interferences from saturation of the signal below 5400 cm^−1^ and the absence of spectral features above 9000 cm^−1^ (data not shown). However, the strong water band at 7000 cm^−1^ displays significant variations according to % *w*/*w* added water concentrations in LALP NADES (Figure 4), while the 9000–8000 cm^−1^ region is less affected by water content in samples. Similar to the case of the ATR-IR spectra, the NADES features in the region 6000–5600 cm^−1^ have an anticorrelated relationship to those of the water, although it is less obvious because they sit on the edge of the strong and varying water feature at 5200 cm^−1^.

The RMSECV of 0.35 +/− 0.08% *w*/*w* added water and the R^2^ value of 0.9982 +/− 0.0029 obtained from the PLSR analysis highlight the strong correlation between spectral variations in NIR-B spectra and % *w*/*w* added water concentration (Figure S2A). Here, three LVs were selected based on the RMSECV plot constructed from the validation sets, shown in Figure S2B. The regression coefficient (Figure 5a) highlights the positive contribution of water with the broad band with a maximum value of 6960 cm^−1^. The negative features at 5800, 5896, 5956 and 5764 cm^−1^ (first overtone C-H stretching) [80,81] correspond to mixed contributions of L-Proline (Figure 5b) and Levulinic Acid (Figure 5c) constituents of LALP NADES. Similar to ATR-IR, the quantification of water in NADES is based on spectral contributions from both the water and NADES constituent components, although, in NIR-B, the relative contribution of the water is significantly higher.

#### 3.1.3. Handheld NIR Spectroscopy (NIR-H)

**Figure 6:**
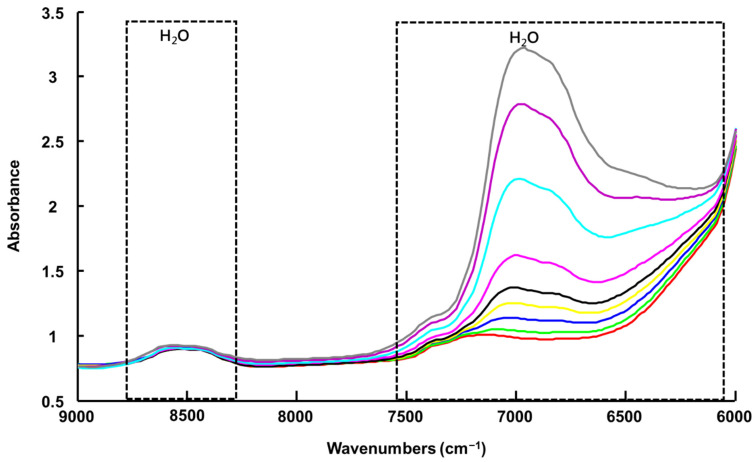
Mean raw NIR-H spectra collected from LALP. Concentrations for added water (% *w*/*w*) are respectively ~ 0% (red), 0.99% (green), 2.4% (blue), 4.7% (yellow), 6.9% (black), 9.1% (magenta), 16.7% (cyan), 23% (purple) and 28% (grey).

For consistency with the results presented in Figure 5, the spectra collected via NIR-H have been cut to 9000 cm^−1^, enabling observation of the intense water contribution, with a maximum value of 6960 cm^−1^, without interference from non-informative spectral windows. Unfortunately, the spectra start at 6000 cm^−1^, which does not allow observation of the contribution from the LALP NADES compounds in the 6000–5500 cm^−1^ window (Figure 6).

The RMSECV value of 0.36 +/− 0.10% *w*/*w* added water and R^2^ equal to 0.9988 +/− 0.0010 (Figure S3A) using six latent variables (Figure S3B) are, however, close to the values obtained via NIR-B, suggesting the smaller spectral window does not have great importance in the construction of the predictive model.

The regression coefficient for NIR-H shown in Figure 7a has strong similarities with the regression coefficient obtained with the NIR-B device, with an intense broad feature corresponding to water between 7270 and 5990 cm^−1^. Only water is observed in the regression model with no features assigned to NADES constituents L-Proline (Figure 7c) and Levulinic Acid (Figure 7d).

#### 3.1.4. Benchtop Raman Microscope (Raman-B)

**Figure 8:**
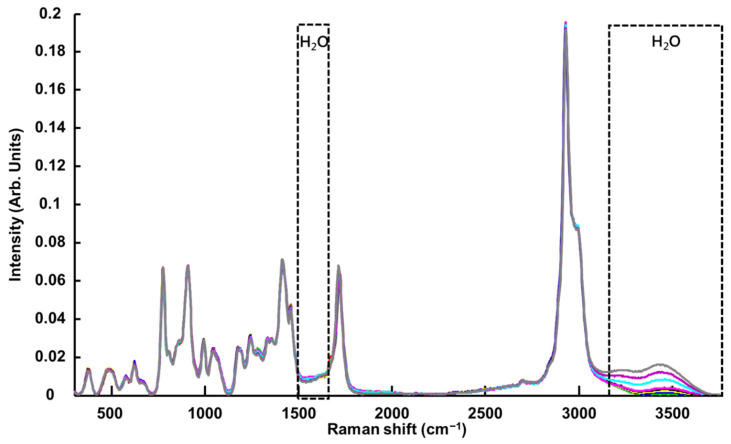
Mean RBVN Raman-B spectra collected from LALP. Concentrations for added water (% *w*/*w*) are respectively ~ 0% (red), 0.99% (green), 2.4% (blue), 4.7% (yellow), 6.9% (black), 9.1% (magenta), 16.7% (cyan), 23% (purple) and 28% (grey).

**Figure 9:**
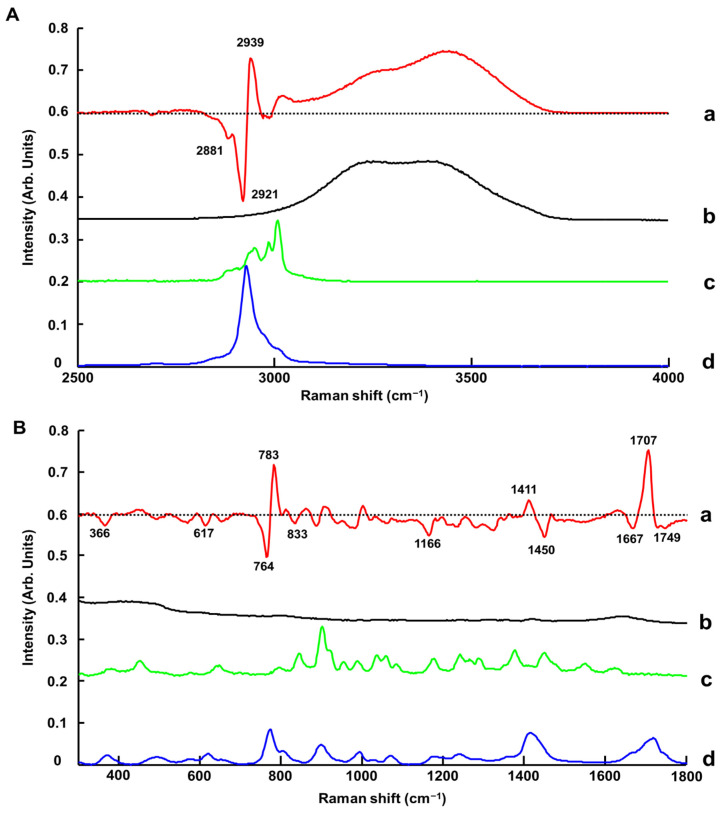
First regression coefficient from PLSR (a), water spectrum (b), L-Proline (c) and Levulinic Acid (d) in the 2500–4000 cm^−1^ spectral range (**A**) and the 300–1800 cm^−1^ spectral range (**B**). Dotted line indicates the zero baseline. Spectra are offset for clarity.

As for ATR-IR, the Raman-B spectrum of H_2_O exhibits a single weak feature at ~1640 cm^−1^ (scissoring bending) and broad OH symmetric and asymmetric stretching modes with two maxima at ~3200 cm^−1^ and ~3400 cm^−1^ [82,83] (Figure 8 and Figure 9A(b),B(b)). The bands observed are in agreement with previous investigations conducted on glycerol-based NADES using a Raman microscope [65] and later using a Raman handheld system [66]. In Figure 8, it is seen that, in the high wavenumber region, variations corresponding to water features can be observed, while in the fingerprint region the strong features from the LALP NADES constituents at ~1480 and ~1700 cm^−1^ obscure the weaker H_2_O scissoring bending. In general, the relative contributions of water vs. the NADES constituents to the Raman-B spectrum are notably lower, and while the effect of the systematic variation of water in the high wavenumber region is apparent, the anticorrelated effect on the NADES constituent contributions is not as apparent.

PLSR performed on raw data resulted in RMSECV = 0.80% *w*/*w* added water (R^2^ = 0.9900); therefore, to improve the quantitative performance of Raman-B, the data were further pre-processed by application of RB and VN. The RMSECV plot (Figure S4B) indicates the PLSR analysis can be performed using five latent variables, delivering RMSECV of 0.43 +/− 0.11% *w*/*w* added water and R^2^ of 0.9977+/− 0.0033 (Figure S4A). From this point forward, only the results from RBVN spectra are presented and discussed.

Figure 9A(a) presents the regression coefficient for the PLSR analysis. An intense and broad positive feature is observed in the spectral range 3060–3700 cm^−1^, specifically assigned to the contribution of water in pre-processed data (Figure S5A). Positive and negative features are observed between 2855 and 3040 cm^−1^.

It is observed in the spectra that a peak shift occurs from 2929 cm^−1^ to 2931 cm^−1^ with increasing % added water concentrations (Figure S5B). Therefore, the intense first derivative-like line shape, observed with a negative feature at 2921 cm^−1^ and a positive feature at 2939 cm^−1^, can be attributed to C-H symmetric stretching vibration of Levulinic Acid (Figure 9A(d)) [84].

In the fingerprint region between 1650 and 1790 cm^−1^, the contribution from Levulinic Acid is evidenced by a double derivative-like line shape that is due to broadening of C=O stretching at 1714 cm^−1^ [84], when water content increases in LALP NADES (Figure S5C). It results in the 1707 (positive), 1667 (negative) and 1749 cm^−1^ (negative) peaks (Figure 9B(a)). Notably, the maximum shifts from 1720 to 1715 cm^−1^. The positive shoulder between 1650 and 1680 cm^−1^ results from the weak contribution of L-Proline (Figure 9B(c)). In the 1380–1500 cm^−1^ region, the positive feature at 1411 cm^−1^ (CH twisting vibration) corresponds to Levulinic Acid [84] and the negative feature at 1450 cm^−1^ (CH_2_ in plane bending vibration) corresponds to L-Proline [79]. Finally, in the 740–801 cm^−1^ region, there is also a first derivative-like line shape feature that is due to the C-C stretching vibration of Levulinic Acid at 772 cm^−1^ [84] that shifts to 778 cm^−1^ with increasing % added water concentration (Figure S5D).

Other weaker features are observed in the fingerprint region at 833 cm^−1^ (CH_2_ rocking vibration), at 1166 cm^−1^ (assigned to L-Proline) [79] and at 617 cm^−1^ (C-C stretching vibration, assigned to Levulinic Acid) [84]. Further features in the fingerprint region cannot be assigned and could also be the result of subtle modifications in the spectra caused by varying water concentrations in NADES. It has been reported in the literature that molecular interactions can be affected by water concentrations in NADES, associated with a weakening of the hydrogen bonding between HBD-HBA, H_2_O-HBD and H_2_O-HBA, which can be observed by Raman spectroscopy [85,86,87]. However, the OH scissoring bending band from water at ~1633 cm^−1^ is weak compared to the other bands and, due to the overlap with the L-Proline band at 1624 cm^−1^, its contribution to the PLSR model is not significant. Ultimately, the PLSR model constructed from the Raman-B data encompasses both the contribution of water, limited to the high wavenumber region, and also numerous bands originating from the NADES compounds.

#### 3.1.5. Overview of PLSR Cross-Validation Results

Table 1 summarises the outcomes for the cross-validation using SET_02 and SET_04 (n = 18 samples). For the purpose of this study, a random split of the data has been preferred to challenge the data analysis and observe the variability in RMSECV and R^2^ when different random combinations of samples are selected as training and validation sets. The selection of optimal latent variables (LVs) is based on the lowest RMSECV value calculated from the 100-fold iteration of cross-validation values, and the values given in Table 1 are mean +/− standard deviation (SD) calculated from those iterations. R^2^ values are all above 0.99, the highest being obtained for ATR-IR with 0.9990 +/− 0.0016 and the lowest for Raman-B with 0.9977 +/− 0.0033. It is observed that SD remains low for all techniques, independent of random combinations of samples selected for training and validation sets, highlighting the reproducibility of analysis performed. The coefficient of determination achieved in regression models for the four techniques demonstrates there is a correlation between variations in spectral features and water concentrations in LALP NADES (Figures S1A, S2A, S3A and S4A).

Compared to R^2^, RMSECV values display more pronounced differences, the lowest equal to 0.27 +/− 0.17% *w*/*w* added water concentration for ATR-IR and the highest being ~2 times higher, at 0.43 +/− 0.11% *w*/*w* added water concentration, for Raman-B. The two NIR systems, as might be expected, have similar performance of 0.35 +/− 0.08% *w*/*w* added water concentration (NIR-B) and 0.36 +/− 0.10% *w*/*w* added water concentration (NIR-H), respectively. Figures S1A, S2A, S3A and S4A show that, for each technique, a linear regression can be achieved by cross-validated PLSR. However, RMSECV and R^2^ already demonstrate that those techniques perform differently when constructing predictive models. While IR and NIRS are highly sensitive to water in the samples, it is observed that the Raman-B technique performs slightly less well, most probably due to the weaker relative contribution of water to the spectra collected.

Plots presenting RMSECV as a function of the number of latent variables display different behaviours (Figures S1B, S2B, S3B and S4B). For ATR-IR, the RMSECV plot starts at 0.81% *w*/*w* added water and decreases to reach the lowest value of 0.27% *w*/*w* added water at four LVs, then starts to increase gradually until the model stabilises at eight LVs with RMSECV approximately equal to 0.30% *w*/*w* added water (Figure S2B). For NIR techniques, three LVs were selected for NIR-B, but the optimal number appeared to be six LVs for the handheld device (NIR-H). However, for both, RMSECV values decreased in the first few LVs then increased up to 20 LVs (Figures S2B and S3B). Moreover, it is observed that standard deviation gradually increases with number of LVs, indicating that noise is included in models, leading to a loss of accuracy and reproducibility. For Raman-B, the optimal number of LVs was found to be five. Looking at the RMSECV plots, it starts at 1.11% *w*/*w* added water, drops gradually to reach its lowest value 0.43% *w*/*w* added water at five LVs, increases slightly with six LVs to 0.44% *w*/*w* added water, and then finally returns to a stable plot with ~0.44% *w*/*w* added water (Figure S4B).

### 3.2. Comparison of Prediction for % w/w Added Water Concentration in Test Sets

Figure S6 presents PLSR plots obtained from samples used as unknowns to be determined from the test set (i.e., SET_01, SET _03 and SET_05) and Table 2 summarises R^2^ and RMSEP values. In Figure S6, it can be seen that a few data points could be considered as outliers. However, in this study, analysing the data as a whole while not removing any samples has been preferred in an attempt to optimise the fitting achieved. R^2^ values for the test sets exhibit a similar pattern for all instruments, and they are all above 0.99. The highest is achieved with ATR-IR (0.9993 +/− 0.0004% *w*/*w* added water) (Figure S6A) and the lowest with Raman-B (0.9955 +/− 0.0016% *w*/*w* added water) (Figure S6D), which is consistent with RMSECV values observed previously. RMSEP values display significant variations between techniques, with ATR-IR performing the best (0.27 +/− 0.08% *w*/*w* added water) (Figure S6A), NIR-B (Figure S6B) exhibiting a value ~2 times higher (0.56% *w*/*w* added water +/− 0.03), NIR-H (Figure S6C) ~2.4 times higher (0.68 +/− 0.08% *w*/*w* added water) and Raman-B (Figure S6D) ~2.4 times higher (0.67 +/− 0.11% *w*/*w* added water). Compared to RMSECV, the difference in RMSEP values between the two NIR systems is more pronounced, the value for NIR-H being ~22% higher than that for NIR-B.

For the purpose of this study, samples used as unknowns to be determined are consistent, i.e., SET_01, SET_03 and SET_05, while samples in the calibration/validation sets are randomly picked from SET_02 and SET_04 using a 100-fold iteration loop.

To more precisely assess performance in relation to the predictions achieved, percent relative errors between the prepared added % *w*/*w* water concentration and the predicted % *w*/*w* water concentration was calculated, and the results are presented in Table 3.

For ATR-IR, the % relative errors range from 0.02% to 14.84%, with mean % relative error equal to 2.58%. The % *w*/*w* added water concentrations were determined with less than 5% relative error for 22 out of 24 samples analysed. The % relative error values above 5% are: 14.84% for C2 (0.99% *w*/*w* added water) and 9.17% for C3 (2.44% *w*/*w* added water) from the test set SET_01. Previously published studies [64,65] reporting water quantification with ATR-IR spectroscopy for three NADES systems (betaine/glycerol: BG, choline chloride/glycerol: CCG and glucose/glycerol: GG) with systematically varying % water concentration (0–40%) delivered RMSEP values of 0.74% *w*/*w* added water, 1.53% *w*/*w* added water and 1.16% *w*/*w* added water for BG, CCG and GG, respectively [64]. The mean percentage relative error was 4.03% *w*/*w* for CCG, 4.08% *w*/*w* for GG and 1.95% *w*/*w* for BG [64]. PLSR results for LALP NADES delivered a lower RMSEP value and lower mean relative errors compared to the CCG and GG NADES previously studied. Glycerol exhibits strong features in the high wavenumber region of ATR-IR spectra that overlap with the water band and partially hide spectral variations [64]. For LALP NADES, neither L-Proline nor Levulinic Acid have contribution in the high wavenumber region; therefore, modifications in this part of the spectrum are more specifically correlated to water concentration, resulting in the better PLSR analysis outcome achieved in the present study.

Results obtained from NIR-B (Table 3) present % relative errors ranging from 0.50% to 20.85%, with a mean equal to 5.13%. The % added water concentrations were determined with less than 5 % relative error for 16 out of 24 samples. The highest values are 20.85% for C2-SET_03 (0.99% *w*/*w* added water), 13.88% for C5-SET_05 (6.97% *w*/*w* added water) and 13.71% for C6-SET_05 (9.09% *w*/*w* added water). In comparison, the NIR-H displays % relative errors over a shorter range (0.88% to 17.21%), although the mean of 6.23% is slightly higher. The difference is explained by the lower number of samples determined with less than 5% relative error (13 out of 24) and the higher number of samples determined with % relative error above 10% (n = 5). The highest % RE values above 10% are 16.99% for C2_ SET 01 (0.99% *w*/*w* added water), 10.71% for C5_SET 01 (6.98% *w*/*w* added water), 12.77% for C2_SET 03 (0.99% *w*/*w* added water), 17.21% for C2_SET 05 (0.99% *w*/*w* added water) and 16.93% for C5_ SET 05 (6.98% *w*/*w* added water).

The Raman-B (Table 3) predictive model provides % relative errors ranging from 0.07% to 20.87%, with a mean equal to 6.75%. The water concentrations were determined with less than 5% relative error for 11 out of 24 samples. Six samples were determined with % RE below 10%. The highest % RE values above 10% are 16.34% for C2_SET 01 (0.99% *w*/*w* added water), 10.44% for C3_SET 01 (2.44% *w*/*w* added water), 19.38% for C2_SET 03 (0.99% *w*/*w* added water), 20.87% for C3_SET 03 (2.44% *w*/*w* added water), 13.27% for C4_SET 03 (4.76% *w*/*w* added water) and 12.14% for C2_SET 05 (0.99% *w*/*w* added water). These values are comparable to both NIR systems, with the mean error and the number of samples above 10% comparable to the NIR-H system while maximum error, 20.87%, is close to the NIR-B (20.85%). A previous study reporting water quantification for three NADES systems (BG, CCG and GG) with macro-Raman spectroscopy [65] delivered RMSEP values of 0.34% *w*/*w* added water, 0.47% *w*/*w* added water and 0.74% *w*/*w* added water for BG, CCG and GG, respectively. The mean percentage relative error was 1.45% *w*/*w* for CCG, 1.18% *w*/*w* for GG and 1.19% *w*/*w* for BG [65]. Unlike IR, the PLSR results for LALP NADES with Raman delivered a higher RMSEP value and higher mean relative errors compared to the CCG, BG and GG NADES previously studied. In glycerol-based NADES models, PLSR was based on the contribution of water features at high wavenumbers and fingerprint regions, as well as NADES features that inversely correlated to the PLSR prediction model [65]. However, with LALP NADES, the PLSR is based on water features only in the high wavenumber region, while in the fingerprint region the spectral changes observed are limited to band shifts that are related to molecular interaction between water and LALP constituents; therefore, the sensitivity of PLSR analysis is affected.

### 3.3. General Discussion

NIRS, MIRS and RS are well established as rapid, label-free and molecular specific techniques for quantitative analysis [88]. In the context of determining water concentration in LALP NADES, the direct analysis of samples without pre-analytical steps and the absence of requirements for any solvents or consumables are indisputable advantages compared to Karl Fisher titration (considered the gold standard). While all techniques studied can deliver an accurate quantification of water in NADES, selecting the most suitable technique depends on a number of criteria. The intrinsic water content of such NADES is >1% *w*/*w*, and the high sensitivity of the gold standard KF titration technique is therefore not required. Spectroscopic analysis offers the benefits of reagent-free and therefore greener techniques, which are potentially field deployable, in the industrial environment.

For example, ATR-IR spectroscopy delivered the best accuracy for the quantification of % *w*/*w* added water in LALP NADES. The PLSR coefficients in Figure 3A(a),B(a) highlight the spectral features of both water and LALP NADES constituents over the full 4000–400 cm^−1^ range, which contributes strongly and plays a key role in constructing a reliable predictive model. The procedure for data acquisition only requires a drop of sample to be deposited onto the ATR crystal to generate highly reproducible spectra. The ATR crystal is relatively small, ~1.8 mm in diameter, enabling collection of high-quality spectra with minimal sampling requirements. Although the technique is user friendly and is simple to operate, samples have to be withdrawn for analysis and, for conventional instruments, analysed one by one. Hence, the procedure for cleaning the ATR crystal, recording a new background and depositing the next drop can rapidly become fastidious for routine use, especially for large cohorts of samples. Recent innovative development of automated ATR sampling as plate readers (AutoATR, Pikes technologies) holds promise for future high throughput analysis that could greatly increase the workflow for spectral acquisition from multiple samples with ATR-IR, overcoming one of the main drawbacks of the technique. ATR-IR probes are not yet commonly used for in situ analysis directly in containers, although a few studies have reported encouraging results for monitoring chemical processes [89,90]. Interestingly, most chemistry or physics laboratories already have ATR set-ups available, and the transfer of the technique to water quantification in NADES is therefore accessible to many researchers in the field. In the current study, a research grade ATR-IR spectrometer that costs roughly EUR 40,000–50,000 has been used, but more affordable systems for routine analysis can be purchased below EUR 20,000.

Acquisition of NIR spectra with the NIR-B and the NIR-H devices was performed in transmission mode in 1 mL glass vials due to the transparent nature of LALP NADES; therefore, withdrawal of samples is also necessary. Using glass vials allows samples to be prepared and sealed until analysis, and there is no cleaning of a crystal or other part of the system between samples; hence, the workflow is improved compared to that of ATR-IR. To reduce consumption of consumables, vials can be washed and reused easily. In this study, a benchtop NIR system (NIR-B) that costs more than EUR 100,000 has been compared to a low-cost handheld device (NIR-H) that can be acquired for ~EUR 2000. Despite technical differences, such as spectral range and spectral resolution, it has been found that both systems performed similarly in terms of R^2^ (0.9969 and 0.9984, respectively) and RMSEP (0.56 and 0.68% *w*/*w* added water, respectively), highlighting that cost-effective alternatives can be developed for monitoring water in NADES. The NIR spectrum for LALP NADES shows strong water features at 6960 cm^−1^ and spectral features for NADES constituents appear between 6000 and 5400 cm^−1^ (Figure 4). As shown in the regression coefficient from both NIR devices, the water feature is enough to build accurate models (Figures S2A and S3A), suggesting that the performance of most commercial NIR systems will be comparable because they all include the water band in the spectral ranges analysed.

Raman spectra show relatively weak features of water compared to infrared techniques (Figure 9). However, the PLSR coefficient from Raman-B (i.e., analysis performed in quartz cuvettes) delivered a RMSECV value of 0.43% *w*/*w* (R^2^ = 0.9977) and a RMSEP value of 0.67% *w*/*w* (R^2^ = 0.9955), comparable to NIR results (Table 1 and Table 2). ATR-IR and NIR data which have been normalised to the reference (air) spectrum deliver the best outcome, and it was observed that Raman spectra had to be subjected to pre-processing before applying PLSR analysis to yield comparable results. For the data collected with Raman-B, a baseline correction coupled to normalisation (RBVN) delivered the best results. This is also consistent with previous studies published on BG, CCG and GG NADES [65,66]. However, it is important to stress that, in recent years, there have been many improvements in acquisition software, including pre-processing and data analysis through user friendly interfaces. Currently, it is easier to apply multiple data corrections in just a few clicks, making the technique more accessible to non-experts. Therefore, requirements for data pre-processing do not differentiate infrared and Raman spectroscopy, and solely the performance in terms of precision and accuracy should be considered.

Although Raman-B results demonstrate the feasibility of performing quantifications of water in NADES using Raman spectroscopy, research grade Raman microscopes can be as expensive as EUR 100,000–300,000, depending on the options selected. However, more affordable commercial handheld Raman systems can be purchased at prices about 10-fold lower. Most of these devices, which are dedicated to field analyses, have fixed gratings to ensure reproducibility of measurement; therefore, the spectral range covered cannot be adjusted, as is commonly possible with benchtop Raman microscopes. It is critical to collect spectra covering the high wavenumber region for applications to water quantification in NADES (Figure 9). In a previously published study that investigated the quantitative performance of the portable Raman Enspectr R532^®^ (EnSpectr, UK) for the determination of water content in three selected NADES (BG, CCG and GG) [66], analysis performed using the full spectral range delivered mean percentage relative error equal to 2.69% for CCG, 8.11% for GG and 6.61% for BG [66]. Importantly, Raman spectroscopy offers promising perspectives for non-invasive in situ analysis, i.e., withdrawal free. Almost all Raman microscope and handheld systems work in confocal mode, enabling the signal to be collected from samples through containers, especially the transparent glassware commonly found in chemistry laboratories [91]. In such set-ups, the technique measures backscattered light and the laser source therefore does not have to go through the sample, enabling the measurement of larger volumes (i.e., bigger glassware, vials or other containers). The main advantages for in situ analysis are ensuring the integrity of the samples while avoiding contamination and greatly improving the workflow while offering a 100% consumable free alternative.

## 4. Conclusions

This study independently demonstrated the potential of ATR-IR, NIR and Raman spectroscopy coupled to PLSR analysis for the quantification of water content in LALP NADES, such that their relative performances could be compared. ATR-IR delivered the best outcome, with RMSECV = 0.27 +/− 0.17% *w*/*w* added water, RMSEP = 0.27 +/− 0.08% *w*/*w* added water and mean % relative error = 2.59%. The regression coefficient from the PLSR analysis highlights the combined contribution from NADES compounds (L-Proline and Levulinic Acid) and water, which strengthens the reliability of the predictive models constructed. Although the technique requires withdrawal and deposition of the sample on an ATR crystal, it is a realistic approach to introducing more environmentally friendly reagent-free water quantification in NADES. Although NIR-B (RMSECV = 0.35 +/− 0.08% *w*/*w* added water, RMSEP = 0.56 +/− 0.03% *w*/*w* added water and mean % relative error = 5.13%), NIR-H (RMSECV = 0.36 +/− 0.10% *w*/*w* added water, RMSEP = 0.68 +/− 0.08% *w*/*w* added water and % mean relative error 6.23%) and Raman-B (RMSECV = 0.43 +/− 0.11% *w*/*w* added water, RMSEP = 0.67 +/− 0.11% *w*/*w* added water and mean % relative error = 6.75%) exhibited lower accuracy compared to ATR-IR, the results, however, clearly highlighted the suitability of these techniques for water quantification in LALP NADES. These methods offer perspectives for potential in situ analysis directly in containers (without withdrawal of samples) using NIR and Raman immersion probes or non-invasively through containers with confocal Raman spectroscopy. While Karl Fisher titration remains the gold standard for moisture content analysis in samples, greener alternatives are available to support the shift towards environmentally friendly approaches. The results reported are encouraging for the future development of tools that can optimise and monitor NADES-based processes while also being transferable to industry.

## Figures and Tables

**Table 1:** PLSR outcome for the cross-validation (SET_2 and SET_04).

Technique	Pre-Processing	Cross-Validation		
		LV	R^2^ +/− SD	RMSECV+/− SD (% *w*/*w* Added)
ATR_IR	Raw data	4	0.9990 +/− 0.0016	0.27 +/− 0.17
NIR-B	Raw data	3	0.9982 +/− 0.0029	0.35 +/− 0.08
NIR-H	Raw data	6	0.9988 +/− 0.0010	0.36 +/− 0.10
Raman -B	RBVN	5	0.9977 +/− 0.0033	0.43 +/− 0.11

**Table 2:** PLSR results for the test sets (SET_01, SET_03 and SET_05). Results are reported as mean +/− standard deviation.

Technique	Pre-Processing	Test Set	
		R^2^ +/− SD	RMSEP +/− SD (% *w*/*w* Added)
ATR-IR	Raw data	0.9993 +/− 0.0004	0.27 +/− 0.08
NIR-B	Raw data	0.9969 +/− 0.0004	0.56 +/− 0.03
NIR-H	Raw data	0.9984 +/− 0.0002	0.68 +/− 0.08
Raman-B	RBVN	0.9955 +/− 0.0015	0.67 +/− 0.11

**Table 3:** Summary of PLSR accuracy obtained with all techniques.

	Technique	% Relative Error						Mean % RE	% RE min–max Values
		<1	˂2.5	<5	<7.5	<10	>10		
Number of Samples	ATR-IR	7	8	7	-	1	1	2.59	0.02–14.84
	NIR-B	1	7	8	4	1	3	5.13	0.50–20.85
	NIR-H	2	4	7	5	1	5	6.23	0.88–17.21
	Raman-B	4	5	2	4	3	6	6.75	0.07–20.87

## Data Availability

Not applicable.

## Supplementary Materials

The following supporting information can be downloaded at: https://www.mdpi.com/article/10.3390/molecules27154819/s1, Figure S1: A: PLS regression model obtained from cross validation using ATR-IR Raw spectra in the range 4000–400 cm^−1^. B: RMSECV according to number of latent variables obtained from ATR-IR Raw data of training sets. Figure S2: A: PLS regression model obtained from cross validation using NIR-B raw spectra in the range 9000–5400 cm^−1^. B: RMSECV according to number of latent variables obtained from NIR-B Raw data of training sets. Figure S3: A: PLS regression model obtained from cross validation using NIR-H. Raw B: RMSECV according to number of latent variables obtained from NIR-H Raw data of training sets. Figure S4: A: PLS regression model obtained from cross validation using Raman-B. B: RMSECV according to number of latent variables obtained from Raman-B RBVN data of training sets. Figure S5: Zooming of mean spectra collected from LALP benchtop Raman RBVN preprocessed spectra in range A: 3100–3700 cm^−^^1^, B: 2880–3040 cm^−^^1^, C: 1650–1760 cm^−^^1^ and D: 740–800 cm^−^^1^**.** Concentrations for added water (% *w*/*w*) are respectively ~ 0%(red), 0.99% (green), 2.4% (blue), 4.7% (yellow), 6.9% (black), 9.1% (magenta), 16.7% (cyan), 23% (red) and 28% (green). Figure S6: PLS regression models obtained from test sets for ATR-IR (A), NIR-B (B), NIR-H (C), Raman-B (D).

## Sample Availability

Samples of the compounds are not available from the authors.
