# Supplementary material for: Comparison of Vibrational Spectroscopic Techniques for Quantification of Water in Natural Deep Eutectic Solvents
Source: Molecules. 2022 Jul 27;27(15):4819. doi: 10.3390/molecules27154819 (PMC9370017; doi:10.3390/molecules27154819)
Supplement: Supplementary file 1 [file molecules-27-04819-s001.zip › molecules-1814125-supplementary.pdf]

Comparison of vibrational spectroscopic techniques for quantification of water in Natural Deep Eutectic Solvents

SUPPLEMENTARY DATA

Suha Elderderi[1,2], Pierre-Yves Sacré[3], Laura Wils[4], Igor Chourpa[1], Abdalla A. Elbashir[5,6], Philippe Hubert[3], Hugh J. Byrne[7], Leslie Boudesocque-Delaye[4], Eric Ziemons[3], Franck Bonnier[1,*]

[1] EA 6295 Nanomédicaments et Nanosondes, Faculté de Pharmacie, Université de Tours, 31 Avenue Monge, 37200 Tours, France;suha.elderderimohmedabdelrhman@etu.univ-tours.fr (S.E.); igor.chourpa@univ-tours.fr (I.C.)

[2] Department of Pharmaceutical Chemistry, Faculty of Pharmacy, University of Gezira, P.O. Box 20, Wad Madani 21111, Sudan

[3] University of Liege (ULiege), CIRM, Laboratory of Pharmaceutical Analytical Chemistry, Department of Pharmacy, 15 Avenue Hippocrate, B36, B-4000 Liège, Belgium; pysacre@uliege.be (P.-Y.S.); ph.hubert@uliege.be (P.H.); eziemons@uliege.be (E.Z.)

[4] EA 7502 Synthèse et Isolement de Molécules BioActives (SIMBA), Université de Tours, 31 Avenue Monge, 37200 Tours, France; laura.wils@etu.univ-tours.fr (L.W.); leslie.boudesocque@univ-tours.fr (L.B.-D.)

[5] Department of Chemistry, College of Science, King Faisal University, P.O. Box 400, Al-Ahsa 31982, Saudi Arabia; bashir_gezira@yahoo.com

[6] Department of Chemistry, Faculty of Science, University of Khartoum, P.O. Box 321, Khartoum 11115, Sudan

[7] FOCAS Research Institute, TU Dublin-City Campus, Dublin 8, D08 CKP1, Ireland; hugh.byrne@tudublin.ie

[*]Corresponding author

franck.bonnier@univ-tours.fr

Key words: Label free water quantification, natural deep eutectic solvent, partial least squares regression, attenuated total reflection infrared, Raman spectroscopy, near infrared spectroscopy

3.2.1. ATR-IR spectroscopy

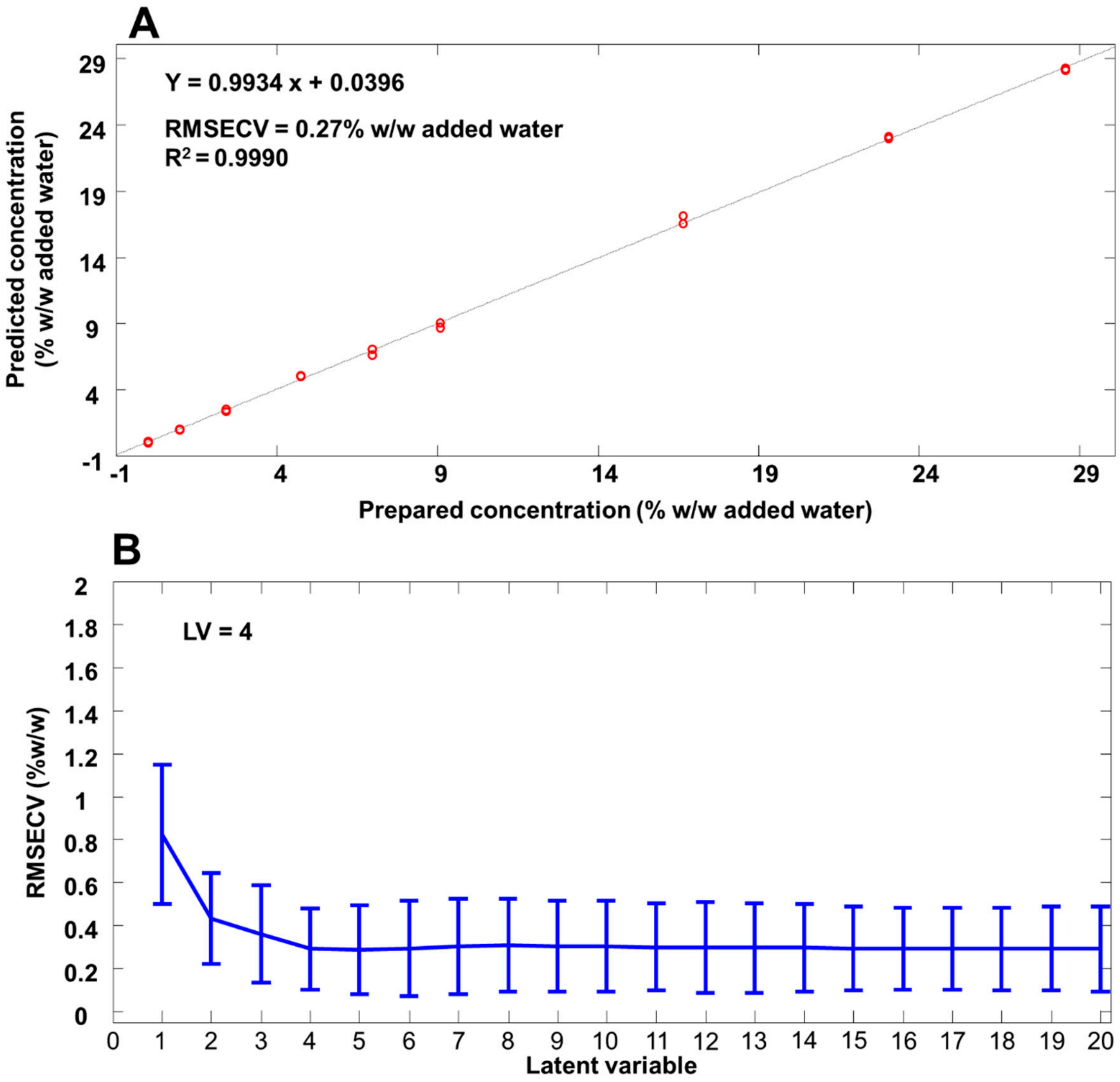

Figure S1 A: PLS regression model obtained from cross validation using ATR-IR Raw spectra in the range 4000-400 cm^{-1}. B: RMSECV according to number of latent variables obtained from ATR-IR Raw data of training sets.

3.2.2 Benchtop NIR spectroscopy (NIR-B)

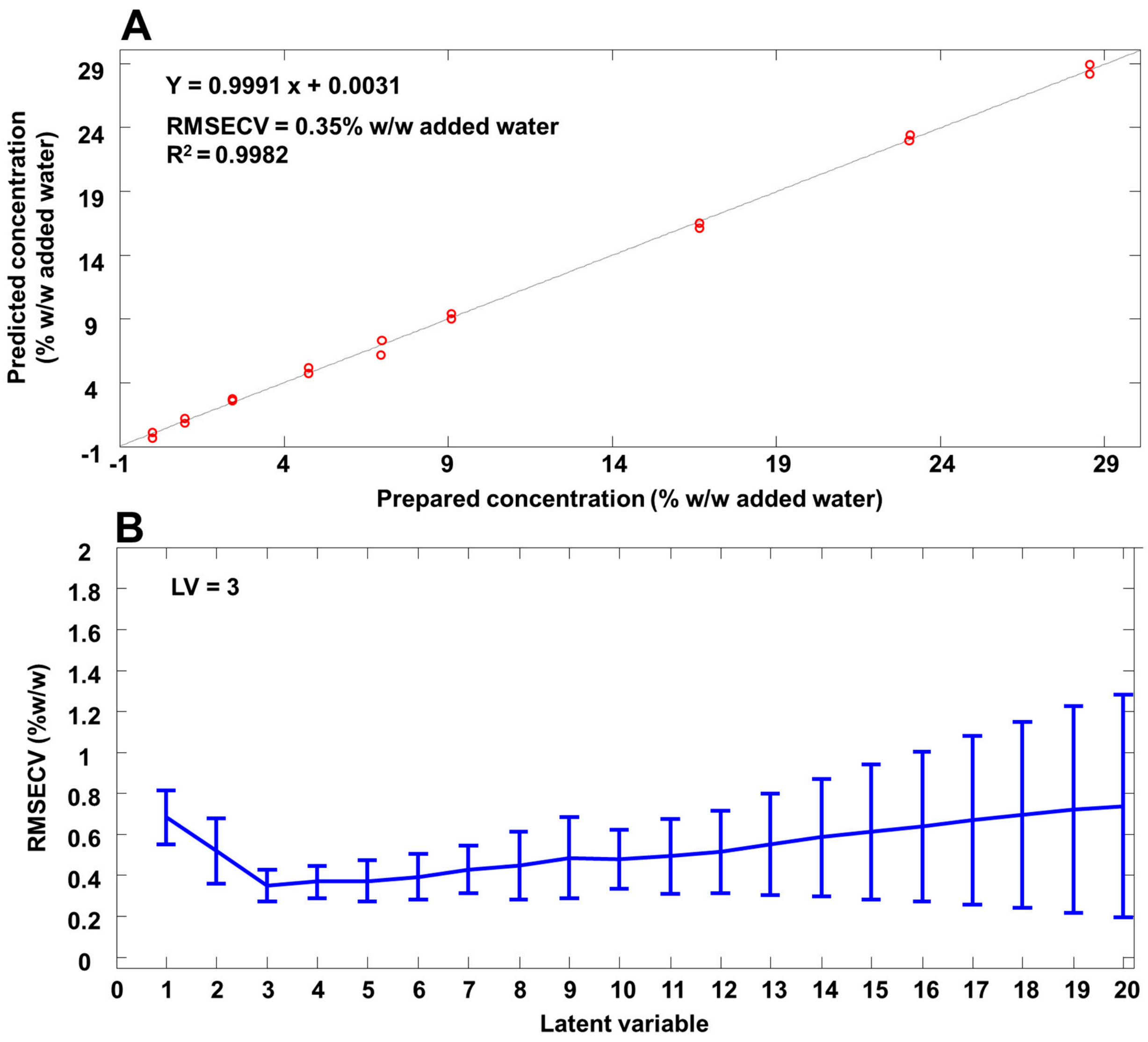

Figure S2 A: PLS regression model obtained from cross validation using NIR-B raw spectra in the range 9000-5400 cm^{-1}. B: RMSECV according to number of latent variables obtained from NIR-B Raw data of training sets.

3.2.3 Handheld NIR spectroscopy (NIR-H)

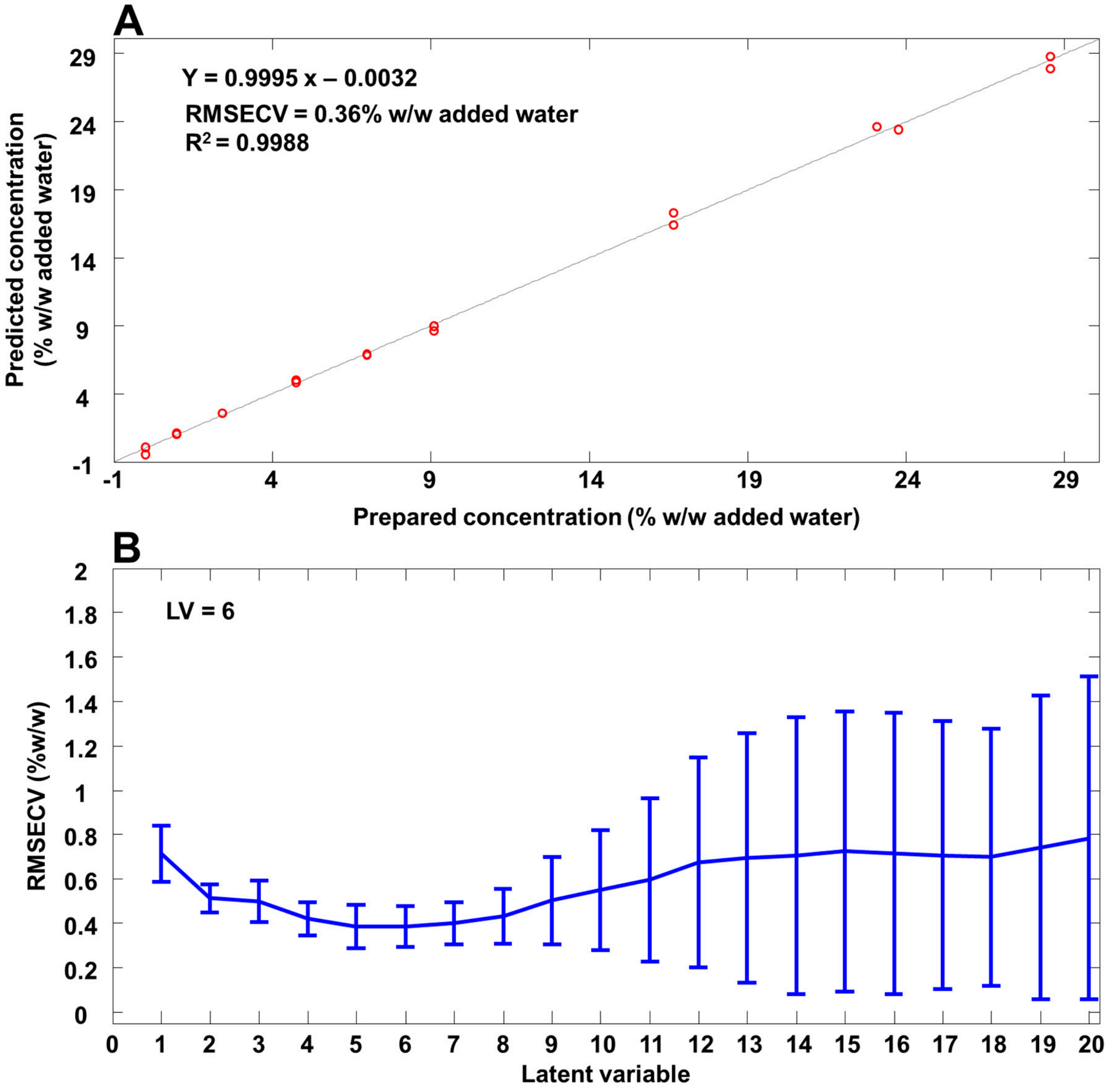

Figure S3 A: PLS regression model obtained from cross validation using NIR-H. Raw B: RMSECV according to number of latent variables obtained from NIR-H Raw data of training sets

3.2.4 Benchtop Raman microscope

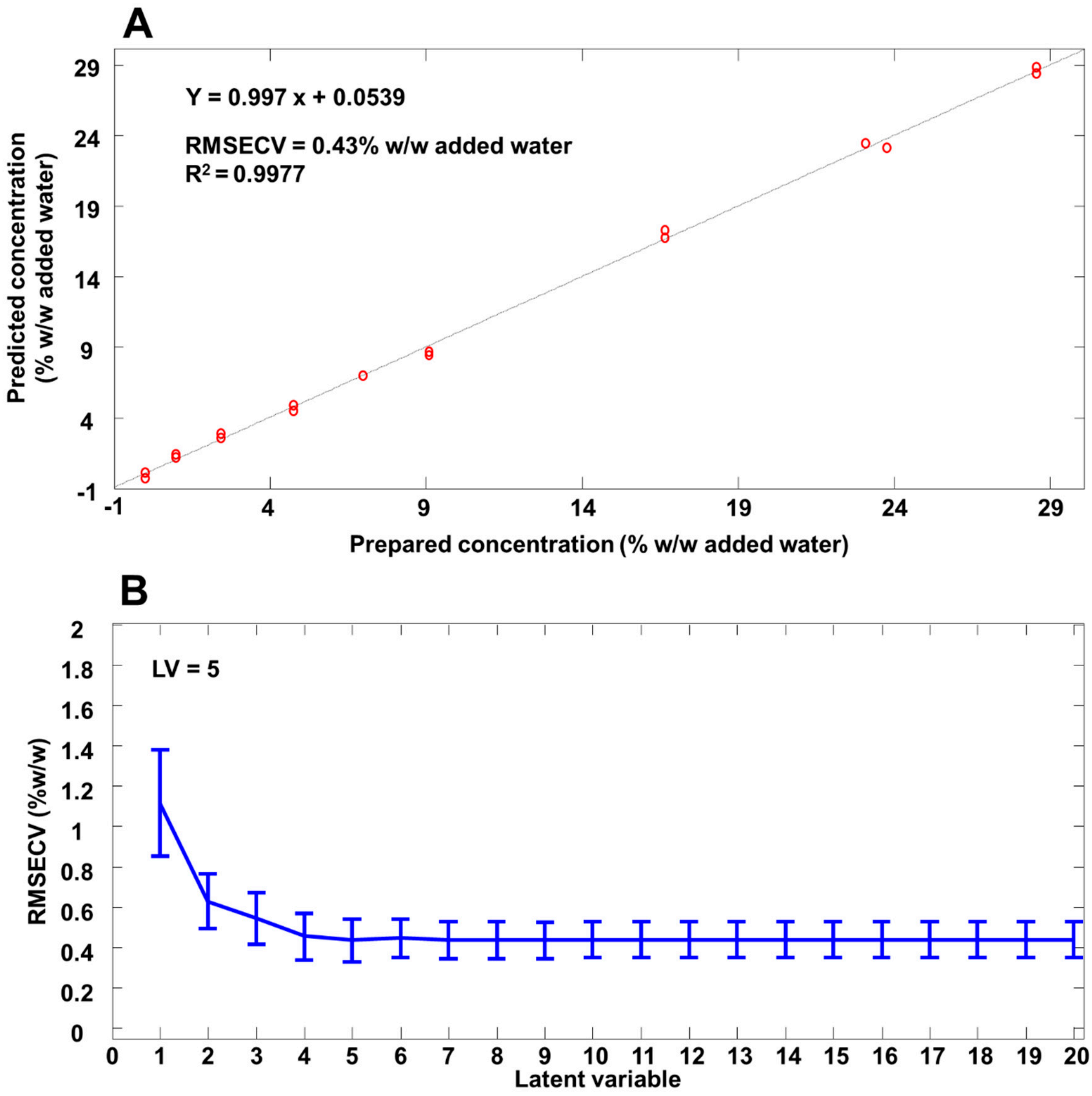

Figure S4 A: PLS regression model obtained from cross validation using Raman-B. B: RMSECV according to number of latent variables obtained from Raman-B RBVN data of training sets.

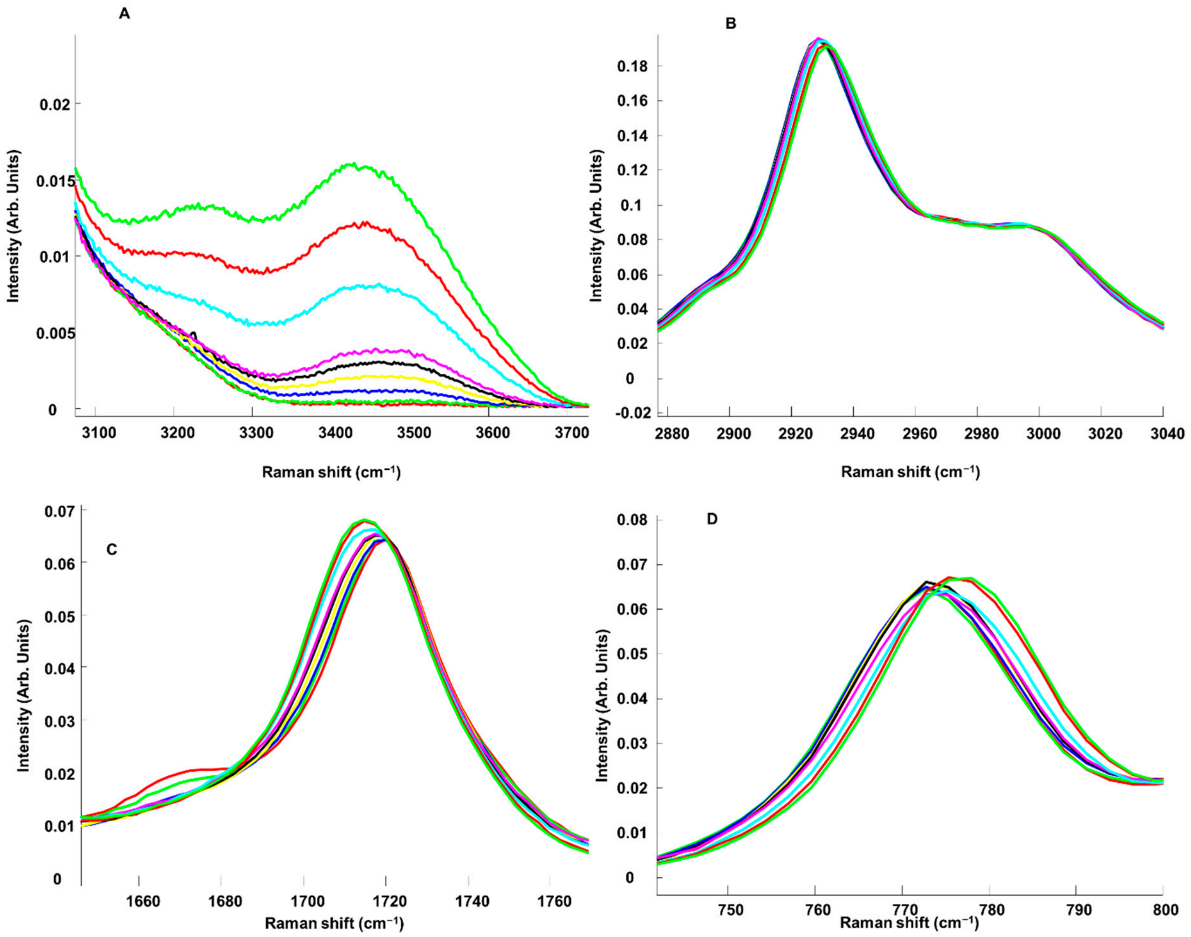

Figure S5 Zooming of *mean spectra collected from LALP benchtop Raman RBVN preprocessed spectra in range A: 3100-3700* cm^{-1}*, B: 2880-3040* cm^{-1}*, C:1650-1760* cm^{-1} *and D: 740-800* cm^{-1}**.** Concentrations for added water (% w/w) are respectively ~ 0%(red), 0.99% (green), 2.4% (blue), 4.7% (yellow), 6.9% (black), 9.1% (magenta), 16.7% (cyan), 23% (red) and 28% (green).

3.2 Comparison of prediction for % w/w added water concentration in test sets

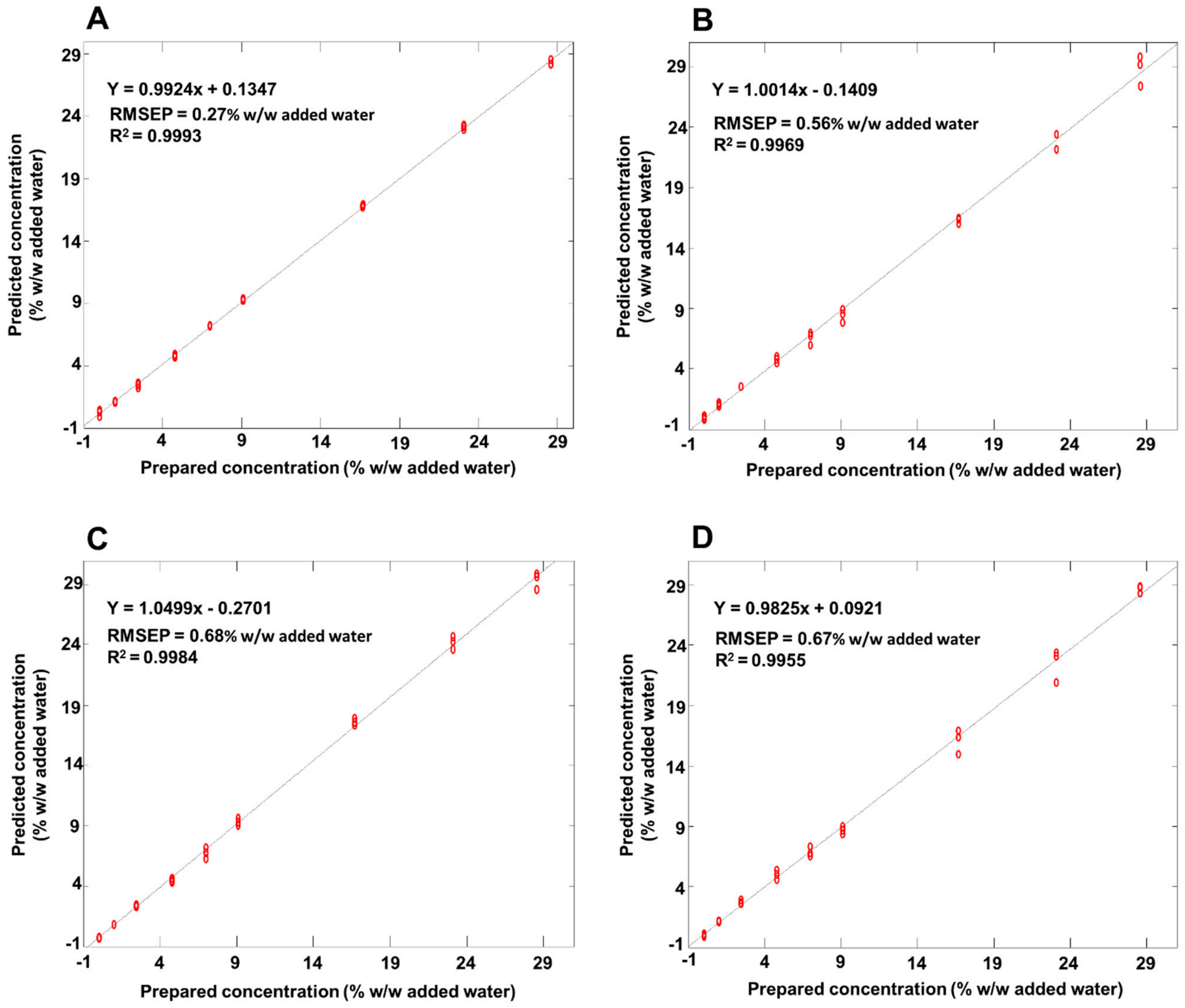

Figure S6 PLS regression models obtained from test sets for ATR-IR (A), NIR-B (B), NIR-H (C), Raman-B (D)
